# Seasick Lungs: How Airborne Algal Toxins Trigger Asthma Symptoms

**Published:** 2005-05

**Authors:** Kris Freeman

Adverse health effects from harmful algal blooms have most frequently been linked to eating fish or shellfish that have accumulated algal toxins. However, people have also suffered asthma-like symptoms after inhaling minute amounts of algal toxins that were aerosolized by waves. Now a research team uses an animal model to gain a better understanding of how exposure to airborne algal toxins causes these symptoms and whether available drugs can be used to prevent or relieve them **[*EHP*** **113:632–637]**.

The researchers focused on toxins produced by a subtropical species of dinoflagellate (*Karenia brevis*) that is a major component of a bloom known as Florida red tide. *K. brevis* produces at least nine types of brevetoxins. When ingested, brevetoxins cause neurotoxic shellfish poisoning, with symptoms that can include numbness, tingling, and gastrointestinal distress. Persons exposed to aerosolized brevetoxins may suffer shortness of breath, sneezing, and other allergy- and asthma-like symptoms. Persons with preexisting airway disease appear most likely to be affected.

To study airborne toxin exposure in a more controlled setting, the research team used a sheep model of asthma. The sheep model used is naturally sensitive to an antigen derived from the roundworm *Ascaris suum*, developing asthma-like symptoms (such as airway constriction) when exposed to this antigen. The sheep therefore can serve as surrogates for persons with asthma. To simulate environmental brevetoxin exposures, these allergic sheep were exposed to crude brevetoxins. These samples contained a variety of brevetoxin species (because multiple types are usually found in Florida red tide) as well as other parts of algal cells (because the toxin is usually released as the algae die and begin to decompose). In addition, the animals were also exposed to two types of purified brevetoxin, presumed to be the primary agent causing respiratory symptoms. In some studies the animals were treated before or after exposure with one of several clinically available medications.

Exposure to crude brevetoxins caused immediate bronchoconstriction in the sheep as evidenced by a twofold increase in airway constriction. This immediate bronchoconstriction was inhibited by 49% (compared with untreated animals) in animals that were pre-treated with budesonide, by 71% in animals pretreated with albuterol, by 58% in animals pretreated with atropine, and by 47% in animals pretreated with diphenhydramine. In addition, bronchoconstriction was quickly reversed in animals that had not been premedicated if they were dosed with albuterol immediately after exposure.

The fact that diphenhydramine, a histamine antagonist, reduced airway symptoms indicates that brevetoxins activate histamine-producing cells, such as mast cells and basophils. Further proof of the involvement of these cells was gained from studies where toxin was injected into the animals’ skin. In these skin tests, the reaction was up to 75% smaller if animals were pretreated with diphenhydramine.

The effectiveness of atropine, an anticholinergic agent, indicates that brevetoxins also activate neural pathways. The cholinergic pathway is involved in the regulation of the neurotransmitter acetylcholine, and is also activated by exposure to organophosphate pesticides.

The researchers also found that bronchoconstriction was reduced by 34% when animals were treated with HOE-140, a bradykinin β_2_ receptor antagonist. This response indicates that, in addition to raising histamine levels, exposure to brevetoxins also increases the level of bradykinin, a protein with effects similar to histamine that has also been linked to asthma symptoms. Asthmatics are more sensitive to bradykinin than are individuals with normal airways, and kinin levels are increased in inflamed airways. This may explain why the researchers found that animals whose airways were already inflamed responded more strongly to brevetoxins. Thus, the increased responsiveness to toxin in persons with preexisting airway disease may be linked to their underlying airway inflammation at the time of toxin exposure.

This research shows that brevetoxins are potent airway constrictors, triggering several physiological pathways. The research has also determined drugs that could mitigate symptoms and serve as rescue medications for persons with severe reactions to brevetoxins.

## Figures and Tables

**Figure:**
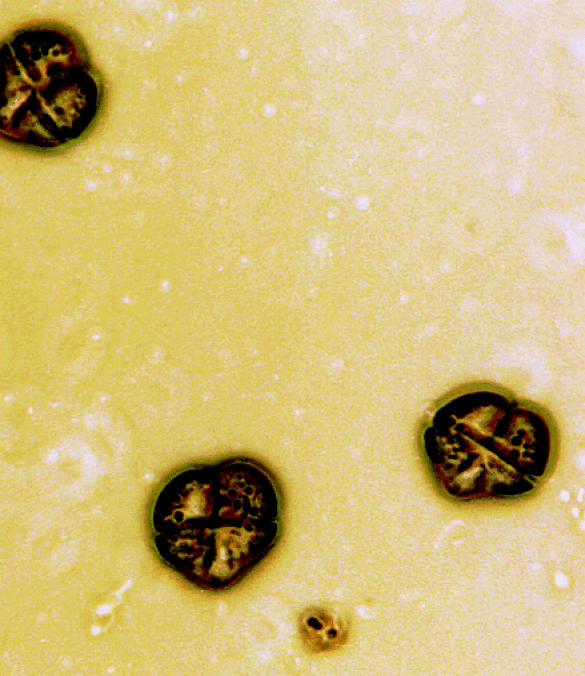
**Waves of illness?** Algal toxins from organisms such as *Karenia brevis* can be aerosolized in sea mist and breathed in by people. A mini-monograph in this issue examines the hazards they pose.

